# Assessment of Levels, Speciation, and Toxicity of Trace Metal Contaminants in Selected Shallow Groundwater Sources, Surface Runoff, Wastewater, and Surface Water from Designated Streams in Lake Victoria Basin, Uganda

**DOI:** 10.1155/2019/6734017

**Published:** 2019-05-23

**Authors:** G. K. Bakyayita, A. C. Norrström, R. N. Kulabako

**Affiliations:** ^1^Department of Sustainable Development, Environmental Science and Engineering, Kungliga Tekniska Högskolan, Brinellvägen, 28 SE-100 44 Stockholm, Sweden; ^2^Department of Civil and Environmental Engineering, Makerere University, Kampala, Uganda

## Abstract

The levels, speciation of elements, and toxicity of selected trace metals as well as other parameters in selected surface water, shallow groundwater sources, landfill leachate, and associated surface runoff in the Lake Victoria basin, Uganda, were studied. The WHO guidelines, Ugandan standards, Canadian guidelines and Swedish EPA were used for assessment. The shallow groundwater was acidic with pH values below 6.5. The pH, dissolved organic carbon, flouride, and sulphate levels for all springs were below the guideline values although 52.8% was contaminated with nitrates while 39% was contaminated with chloride ions. Some surface water samples had levels of major elements, such as iron, chromium, aluminium, and manganese, above the guideline values. Speciation studies showed that 74% of the metal ions was bound to dissolved organic matter in surface water, whereas in landfill leachates, the dominant ionic species was metal hydroxides or fulvic acid bound. Risk analysis based on the Swedish EPA showed varied risks of negative effects in 30%–76% of the sample sites ranging from high to increased risk in surface water, whereas the results from modelling sorption data using the Bio-met tool showed potential risk to toxicity effects of Cu^2+^, Ni^2+^, Zn^2+^, and Pb^2+^ in 15.3%–30.8% surface water samples and 8.3%–62.5% groundwater samples.

## 1. Introduction

The presence of heavy metals and other pollutants in the aquatic systems has become a serious problem for environmental scientists in many developing countries and also for agencies engaged in environmental production [1]. Freshwater resources constitute only about 2.5% of the total volume of water on Earth (∼1.4 million·km^3^), and only about 0.01% (200,000 km^3^) of all the water on Earth is usable freshwater for ecosystems and humans [2]. While the freshwater volume remains generally constant, the population using the water continues to rise, stressing this supply more critically each year [3]. Freshwater is therefore a meagre resource whose quality is very difficult and costly to reverse to pristine and usable condition when it deteriorates [4]. Uganda is a developing country that is home to the source of the River Nile which is the longest river in the world that moves 6,850 km to the Mediterranean Sea from Lake Victoria. Lake Victoria is the second largest freshwater lake in the world with a surface area of 68,800 km^2^ and a volume of 2,760 km^3^ [5]. The Nile river basin covers almost 10% of the total area of the African continent with an area of 3,100 km^2^ [6]. There are eleven countries that make up the Nile River drainage basin, namely, Rwanda, Burundi, Democratic Republic of Congo, Uganda, South Sudan, Sudan, Kenya, Tanzania, Ethiopia, Eritrea, and Egypt. Some of those riparian countries only have a small part of their area within the basin while others are located virtually entirely within the basin [5]. Nile River drainage basin serves the domestic, industrial, and agricultural needs over 150 million people. The Lake Victoria basin, Uganda, forms a bigger part of the upper Nile basin that covers much of Uganda where it serves over 36 million people. There are socioeconomic as well as industrial activities of the urbanizing region with consequential unprecedented anthropogenic contamination of the basin's surface water system which may eventually irreversibly impact the shallow aquifers. The extent of anthropogenic environmental pollution in the developing world is well documented [7]. Geologically, Uganda is dominated by crystalline rocks which are covered by regolith, a layer of the weathered material that varies from rock fragments to well-weathered soil and hardened laterite at the ground surface [8]. The British Geological Survey [8] reported that groundwater is abstracted from both the fractured bedrocks and the weathered regolith. According to Taylor and Howard [9], the regolith aquifer is a reusable resource due to its promising yields and lower cost of development than the deeper groundwater from the basement. However, the commonest groundwater sources in Kampala abstract water from shallow aquifers through protected and unprotected springs. Spring waters are the least mineralised with compositions approaching those of the surface waters [10] which indicate that groundwater in shallow aquifers discharges fast with minimum chemical interactions with aquifer minerals. The shallow aquifers are prone to contamination from anthropogenic sources due to unorthodox handling and management practices for domestic, municipal, and industrial wastes and wastewater. For instance, the unlined landfills for the urban wastes are located in wetlands and pollute the aqueous environment with noxious gases and leachate. Muwanga and Balifaijo [11] revealed that industrial effluents were one of the main sources of pollution in the Lake Victoria basin, Uganda. Kulabako et al. [12] indicated that the quality of the shallow groundwater in periurban areas is a potentially major environmental health problem, and Howard et al. [13] observed that the consumption of contaminated drinking water in the densely populated periurban areas is one of the most significant causes of ill-health. Relatively little data are available on the extent of environmental pollution because there are no agencies charged with the routine monitoring and protection of the environment [14]. Therefore, the assessment of levels and speciation of toxic trace metal contaminants in shallow groundwater, surface runoff, and surface water from selected waterways would reveal the state of environmental health of the shallow aquifers associated with them.

Trace metals are always present in trace amounts in fresh waters from terrigenous sources such as weathering of rocks resulting in geochemical recycling of heavy metal elements in these ecosystems [15, 16], but Sekabira et al. [17] observed that heavy metal pollution of aquatic ecosystems is becoming a potential global problem. Inadequate waste handling and management systems as well as the release of untreated dumpsite leachate and industrial effluent to the environment have significantly contaminated wetlands, surface water, and groundwater which affect the proper functioning of the aquatic ecosystems and endanger aquatic life especially when the pH is low. Studies on an urban open drain Nakivubo Channel have indicated high concentration of toxic trace metals in water and the soils in the near-stream zone that are flooded with contaminated water [18, 19]. Moulodi and Thorsell [20] observed that infiltration is however taking place by localized recharge of different types of wastewater in the periurban areas, which has a clear negative impact on the quality of water resources. The contamination of land and surface water ecosystems with trace metals will continuously threaten the aquifers and shallow groundwater resources for a long time. The environmental health effects of trace metal contaminants are manifested after chronic exposure to low levels or acute exposure to high doses of trace metals. Trace metals are grouped into essential and nonessential trace metals depending on their biological importance. The essential trace metals are those that have known biological roles in living organisms, whereas the nonessential trace metals interfere with the uptake of the essential ones and have known toxicity to life processes. Trace metals such as cadmium, mercury, lead, copper, and zinc are regarded as serious pollutants of aquatic ecosystems due to their environmental persistence, toxicity, and ability to be incorporated into food chains [21]. Essential trace metals such as zinc show toxicity effects when ingested in excess of the optimal levels or in insufficient amounts to bodily needs, whereas nonessential trace metals such as lead and cadmium express toxicity effects when taken in above the tolerable levels.

The major anthropogenic sources of trace metals are combustion of fossil fuels, sewage sludge, and waste dumpsite leachates as well as the wearing of car tyres, brake pads, bearings, radiators [22], mining, smelting, and processing industries. The high concentrations of trace metals are attributed to wastes and industrial effluent deposited in the environment which may result from wearing of machines, smith workshop waste, motor service centres, surface runoff, and industrial chemicals. The main recipient of these metals is the soil, and their fate depends on the chemical and physical properties of the metal or its compounds, soil type, and its organic matter content. Adsorption onto soil particles and soil organic matter causes retention of trace metals and reduces their transmission to the soil water, but when the soil is overladen or when the soil pH is lowered, trace metals' vertical mobility increases and contamination of groundwater occurs through the soil solution. Consequently, the trace metal contaminants may show toxicity effects on fauna since the flora are more tolerant to high levels of trace metals.

The aim of this study was to assess the levels of major and trace elements and establish the speciation of selected trace elements in the aqueous environment of selected shallow groundwater, surface runoff, surface water, landfill leachate, and wastewater in several subcatchment areas in Lake Victoria basin, Uganda. The other objective of the study was to risk assessment for selected trace elements in designated surface water and shallow groundwater sources to inform policy and action on remediation and protection of environmental health. The speciation of trace elements in the aqueous environment will show the distribution of the different metal species amongst metal ions bound to organic species, free metal ions, and inorganic metal ions which would have different effects on environmental mobility, distribution, and life support systems.

## 2. Materials and Methods

### 2.1. Study Area and Sampling Sites

**Figure 1:**
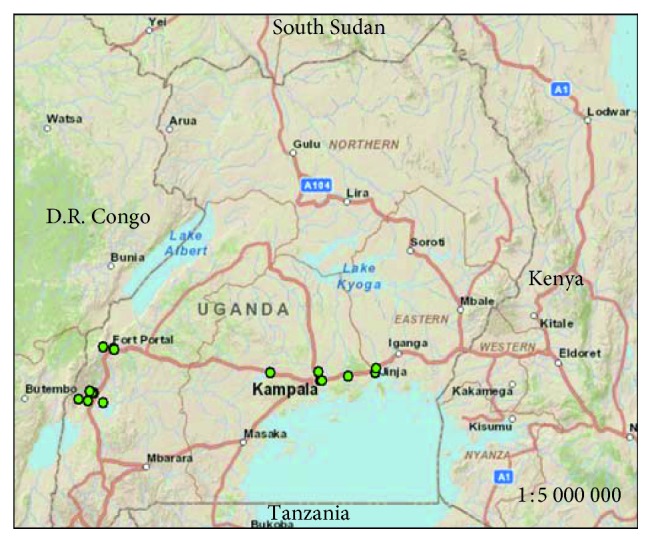
Map of Uganda showing the sampling sites for surface water from urban and rural waterways.

**Figure 2:**
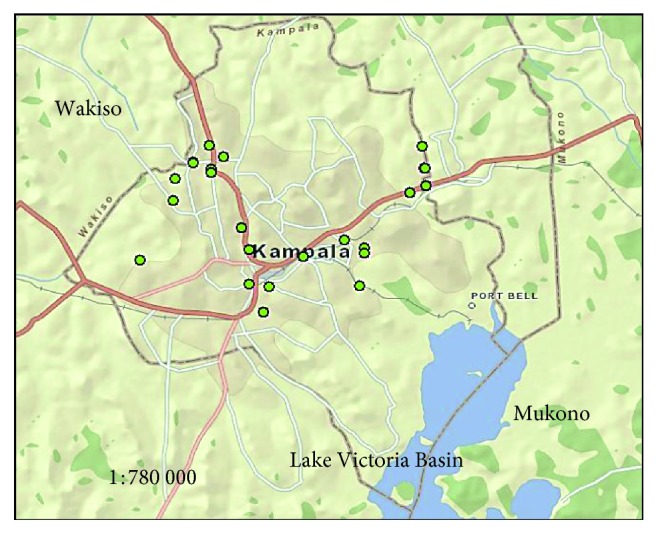
Map of Kampala city showing shallow groundwater and surface runoff sampling sites.

The study area is the Lake Victoria basin, Uganda, with sampling points on streams (Figure 1) and protected springs (Figure 2). Some of the coordinates of the sampling points mapped in Figures 1 and 2 are shown in Table 1. The streams, protected springs, and landfill leachate that were sampled and studied are within in the catchment areas associated with Lake Victoria basin and are located in Kampala Capital City (Figure 2) which is the country's most industrialized business district. The choice of the sampling points in the study area was based on the resident population density and anthropogenic activities in the area, dependence of the population on the shallow groundwater, and surface water sources as well as logistics and time limitations.

### 2.2. Water Sampling

The shallow groundwater from protected springs was drawn as it discharged from shallow aquifers. The sampling of shallow groundwater was done four times, once each year from March 2012 to February 2015. Surface runoff that flowed and settled in the catchment areas associated with the protected springs was trapped in shallow patches from which grab samples were picked thirty minutes after the onset of heavy downpours of the wet season, from September to October 2013. Three grab samples were picked and homogenised before a representative sample was taken for analysis. The surface runoff was sampled because there is a risk of intrusion to the shallow aquifers since during wet seasons the water table rises and increases probabilities of surface water and shallow groundwater mixing. The surface runoff assessment is therefore a basic and meaningful part of hydrological studies. The surface water samples from urban and rural waterways as well as leachate samples from unlined landfills were picked at points 1 m from the peripheral at depths within 0.5 m of the surface. The water samples were collected from the sample sites in 500 mL plastic bottles that had been prewashed thrice using sample water prior to collection. Water samples were filtered through 0.45 *µ*m nonpyrogenic syringe filters and stored in 30 mL polypropylene vials. The pH, temperature, and electrical conductivity were measured at the point of collection of samples. The samples were placed in cooler boxes and transferred to the analytical laboratory.

For streams, the sampling was done twice in June 2014 and February 2015. The plastic bottles used for collection of water samples were cleaned and rinsed twice with the water to be sampled before use. Field measurements of pH, temperature, and electrical conductivity (EC) were measured using a pH Ion meter. The water samples for determination of DOC were filtered into sample bottles using 0.45 *µ*m filters and cooled before being transferred to the laboratory. The water samples were subsampled twice; one sample was filtered and cooled, while the sample for trace metals analysis was filtered and acidified with concentrated nitric acid before being transferred to the analytical laboratory at room temperature (25°C).

### 2.3. Metals, Anions, and DOC Analyses

The acidified water samples were subsequently analysed for trace and major elements using an inductively coupled plasma-mass spectrometer (ICP-MS) (2007, Model: PerkinElmer Elan 9000) with an instrument limit of detection of 0.002 ppm for cadmium and 0.009 ppm for lead, whereas the samples for DOC and anions analysis were evaluated using the Shimadzu TOC-5000 without acidification. The leachate samples were digested using the wet acid digestion procedure. Pretreatment of 50 ml of leachate was done with 2 ml nitric acid, and 5 drops of 30% hydrogen peroxide were added in a closed bottle and placed in a constant temperature water bath at 60°C for 1 hour. The clear supernatant was filtered through 0.45 *µ*m filters into plastic sample bottles and analysed for cations, whereas the samples for DOC and anions analysis were only filtered before analysis. The analyses were done over a period of three years, and mean values of the measurements have been reported. The major anions Cl^−^, NO_3_^−^, and SO_4_^2−^ were analysed at KTH, Stockholm, using a Dionex DX-120 ion chromatograph.

### 2.4. Speciation and Toxicity Studies

The chemical species distribution in organic and inorganic phases would impact on the sorption process since the metal ions in the free ionic state and inorganic state would be available for sorption onto biosorbents and would also be important for toxicity studies. The speciation of selected trace metals was performed with Visual MINTEQ 3.1, and risk assessment of trace metals to aquatic fauna was done by comparison with the Swedish environmental guidelines for selected shallow groundwater and surface water samples. Pb^2+^ and Cd^2+^ ions speciation in selected contaminated surface water and leachate at various conditions of DOC and pH was performed with Visual MINTEQ 3.1 which was originally coded by the U.S. Environmental Protection Agency (EPA) and has been further developed by Gustafsson [23, 24]. For the speciation studies of the metal ions in selected surface water, the Stockholm Humic Model (SHM) was used. The assumption used by the US EPA in setting the Water Quality Criteria in which all DOC in natural waters was considered to be active and organic matter consisted of 90% fulvic acid and 10% humic acid was adopted in these studies. Toxicity studies were done by assessing the environmental risks due to trace elements through modelling using the Bio-met software tool. The Bio-met software tool version 4 is based on calculations from Biotic Ligand Models (BLM) to estimate the potential risk to the aquatic environment posed by copper, nickel, zinc, and lead after considering bioavailability. The basis of biotic ligand modelling is that metal accumulation at the “biotic ligand” is proportional to toxicity and that this accumulation can be predicted by solving the appropriate simultaneous equilibria among the biotic ligand(s) and the dissolved components (aqueous ligands and competing cations) in the exposure water [25]. Data analysis for environmental risks due to trace metals was done using the Swedish environmental protection agency criteria and the bioavailability-based approach through simulations using the biotic ligand model and the Bio-met software tool.

**Figure 3:**
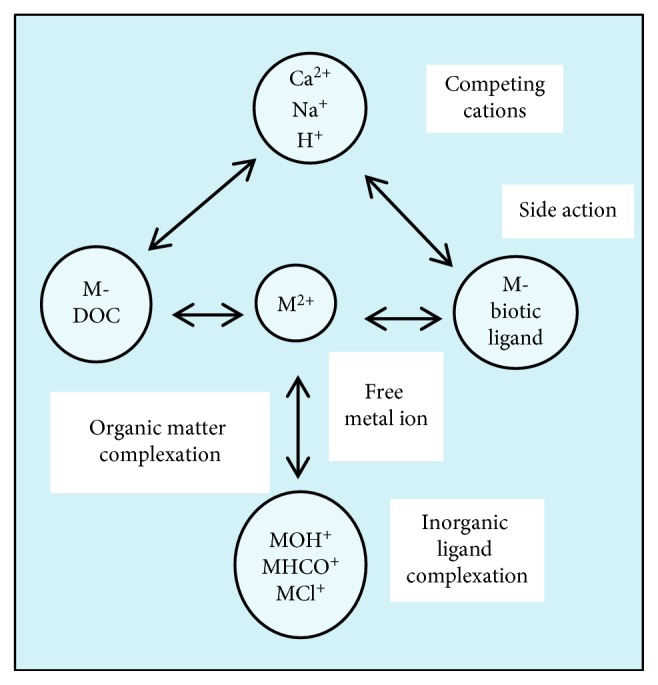
Remodified schematic diagram of the biotic ligand model. M^2+^ is the free metal ion (after [26]).

In Figure 3, a remodified schematic diagram of the biotic ligand model (BLM) has been presented that explains the assumptions and interactions between the inorganic, organic, and biotic ligands in the aqueous media. These interactions are shown in Figure 3 with the free metal ion, M^2+^, and naturally occurring competitive cations, Na^+^, H^+^, Mg^2+^, and Ca^2+^, and abiotic ligands by DOC, CO_3_^2−^, HCO_3_^−^, OH^−^, and Cl^−^, whereas the site toxic action is represented by the biotic ligand or test organism under study. According to the conceptual framework of the BLM, the accumulation of the metal ions at the biotic ligand or greater than a critical threshold concentration of metal ions onto the biotic ligand leads to toxicity effects. BLMs allow chemical and biological interactions to be taken into account and relate, through water chemistry, metal toxicity to a dissolved concentration which can be used in the compliance assessment [27].

## 3. Results and Discussion

The levels of major elements in shallow groundwater, surface runoff, landfill leachate, wastewater, and surface water were analysed and studied with the guidance of WHO guideline values, Ugandan standards, and the Swedish Environmental Protection Agency guideline values [28–30]. According to the WHO guidelines, the results for selected protected springs in Kampala which are shown in Tables 2 and 3 indicate that 58% of the sampled protected springs are contaminated with Fe which exceeds 0.3 mg/l in the range 0.34–1.86 mg/l. This exceedance of the guideline value can be attributed to weathering, and a clear testimony is observed with surface water in the corresponding wetlands having a rusty flimsy layer in slow and stagnant waters. The results for Fe are in agreement with the report by BGS [8] where it was stated that iron was one of the main inorganic groundwater quality problems. Iron is mainly transported in groundwater in the reduced form as Fe^2+^. Therefore, the main source of Fe contamination appears to be background levels in the soils where acidic and redox conditions enhance release of Fe and Mn into soil water. There were only 4% of the springs that had Cr levels above the 0.05 mg/l level.

The protected springs with the elevated Cr levels are located in Nakawa division, and their contamination may be attributed to the existence of paint manufacturing industries in the spring catchment zone. However, 8% of the samples had high levels of Al (0.299–0.360 mg/l) exceeding the 0.2 mg/l level. The contamination of the environment with aluminium may be due to anthropogenic activities besides wear and tear of motor parts since the contaminated sampling points were located in a built environment with paved roads but near a car parking lot. On the contrary, the soils in Kampala were reported to be acidic by Moulodi and Thorsell [20], whereas under acidic conditions, the natural sources of metals may contribute to high levels of aluminium since it is mobilised and solubilized from bedrock and soil into soil water at pH < 5.5. The springs that had levels of K above the guideline value, 10 mg/l, were 33% and had values between 15 and 17 mg/l, whereas 29% had Mn levels above the 0.4 mg/l mark with values ranging between 0.58 and 1.20 mg/l. The BGS [8] report indicates that high concentrations of manganese are a common problem in Ugandan groundwater. The high iron and manganese occurrences have been attributed to the shallow clayey regolith that restrict aeration of underlying aquifers leading to anaerobic conditions to which the high iron and manganese are related [8]. These results for iron and manganese concur with other studies in Uganda by GIBB [31] where iron levels were reported as 0.3–4.9 mg/l; moreover, Taylor and Howard [32] reported iron concentrations up to 45 mg/l and the Mn levels up to 2 mg/l. The water samples that had Ca levels below the required levels for drinking water (20 mg/l) were 95.5%, and with the low pH, it may be a health risk. According to the Ugandan standards, the levels of contamination of the groundwater samples were 20% for Al, 4.2% for Cr, 66.7% for Mn, and 58.3% for Fe, whereas the other major elements were within the Ugandan urban drinking water standards.

Tables 4 and 5 show levels of trace elements in protected springs and percentage springs with levels above and below the guideline values, respectively. For the case of Ugandan standards, all the elements were below the guidelines, whereas for the WHO guideline values, 8.3% of the protected springs had barium levels above the guideline value, and all the other elements were below the guideline values.

For Canadian guideline values, 8.3% of the springs had antimony levels above the guideline value. The contaminated protected springs were located on the peripheral of a busy urban market, car washing bay, and motorcycle service workshop which were suspected to be the sources of contamination.

The protected springs that had elevated levels of trace elements were characterised by nearness to motorways and residential areas where wearing of car parts, surface runoff, and domestic wastewater are assumed to impact the metal levels. The mobility of trace metals increases under acidic conditions with high dissolved organic matter content, but contamination of the acidic shallow groundwater was not alarming due to low dissolved organic matter content. The sampled shallow groundwater from Kampala had elevated levels of trace metals and was not contaminated; nevertheless, groundwater quality monitoring will ensure adherence to the WHO guidelines to inhibit water contamination and imminent trace metal poisoning.

Tables 6 and 7 show the other parameters of water that were studied. The temperature of the water varied but was within the guideline value 25°C; moreover, the pH values were all below the guideline values range of 6.5–8.5. The shallow groundwater is therefore generally acidic with pH < 7.0, whereas acidic water increases the weathering of rocks and release of metals from soil surfaces into soil water.

Acidic water is aggressive to surfaces and has a high capacity to corrode ferrous materials which explain why surface water in the catchment areas exhibits a flimsy rusty layer. On the contrary, Olade [14] stated that groundwater tapped from weathered regolith is acidic which may promote oxidation and corrosion of steel casings and screens. The DOC levels were all below the contamination level (2.0 mg/l). Mobility of trace metals is known to be dependent on the dissolved organic matter content and pH although redistribution of trace metals in groundwater may be affected by other factors such as redox conditions.

The electrical conductivity (EC) values were high and exceeded the guideline value of 1000 *µ*S/cm in almost 13.5% of the shallow groundwater case studies and 21.6% of the surface runoff samples studied which indicated that inorganic species occurred mainly in the ionic form especially in the high population density periurban areas where those particular protected springs were located. Only 5.4% of the sampled spring water had nitrate levels below the Ugandan standard of 10 mg/l, and the rest was highly polluted with 11–125 mg/l which implied that there was enrichment of the shallow groundwater with nutrients from urbanisation and industrialisation activities. Of the sampled shallow groundwater, 52.8% had nitrate levels above the drinking water guideline value of 50 mg/l and 21.6% of them had elevated levels. The Canadian guideline value of 45 mg/l was exceeded by 73% of all sampled springs. These results were in agreement with studies by Taylor and Howard [32], where nitrate levels of 26 mg/l were reported in groundwater, and in another study by Kulabako et al. [35], the mean nitrate values reported in shallow groundwater were 67 mg/l. These high nitrate levels are indicative of nutrient loading of the shallow aquifers which could be attributed to anthropogenic sources such as onsite sanitation, domestic sewage disposal habits, waste dumpsites, and others in the spring catchment areas. All the chloride levels in the spring water samples were below the guideline values although they were elevated and indicated increasing salinity probably due to weathering since the groundwater is acidic; however, these results were similar to the high salinity reported by Kulabako et al. [35] which was 59 mg/l, whereas Moulodi and Thorsell [20] reported 4.6–54.6 mg/l of chloride levels in shallow groundwater. One of the most serious inorganic contaminants reported was fluoride with concentrations above the guideline value, 1.5 mg/l for samples of groundwater in interaction with crater lakes in Uganda [8, 36], although the fluoride levels in shallow groundwater in Kampala were lower than the guideline values, which indicated a difference in sources of fluorine which are of geochemical origin. All the surface runoff associated with the protected springs had high electrical conductivity with 21.6% of them above the guideline value and 27% with elevated levels but below the guideline value of 1000 *µ*S/cm. Weathering, wearing, and corrosion of metals dissolve metals in the wash down and increase the electrical conductivity of surface runoff. About 2.7% of the surface runoff samples were above the Ugandan guideline value of 10 *µ*g/l for cadmium, while 5.4% of the samples were above the Canadian guideline value of 5 *µ*g/l. All fluoride levels were below the Ugandan guideline values and Canadian standards. When the levels of metals in surface runoff were compared to the effluent discharge standards [37], 81.1% of them were above the guideline value of 50 *µ*g/l for lead. As per the Canadian standards [38], 97.3% of the samples had lead levels above the guideline value of 10 *µ*g/l. The suspected source of lead contamination is mainly anthropogenic activities since there are a lot of scrap yards, motor service centres, and metal workshops in addition to wearing of parts of vehicles that may contribute to the elevated levels. Otherwise continued discharge of untreated surface runoff, wastewater, and leachate will negatively impact the shallow groundwater when the soil water is laden during the rainy season; the surface water interacts with the groundwater in shallow aquifers without reacting with the soil chemicals and thence groundwater contamination.

Table 8 shows the levels of major elements and other parameters in surface water, wastewater, and landfill leachate from selected sources in the Lake Victoria basin.  Table 9 shows a summary of the levels above and below the guideline values, respectively. The pH for all the sampled sources was within the limits of the WHO guideline values (6.5–8.5). The electrical conductivity values for 53.3% of the surface water, surface runoff, and landfill leachate samples were higher than the guideline value of 1000 *µ*S/cm, whereas samples from the hot springs and municipal landfills leachate were 3–6 times higher than the WHO guideline value. These values could be due to mobilisation of conducting ions during the decay processes of landfills and thermal mobilisation of ions as hot water rises up through mineralised soils in hot springs; moreover, the salt lake has a rich matrix of conducting ions. The mine tailings from an old copper mine had higher EC values that were attributed to copper contamination of water. The temperatures of surface water sources were within the guideline values. The exceedance above the WHO guideline values for major elements in surface water was 33.3% for sodium, 13.3% for magnesium, 33.3% for aluminium, 53.3% for potassium, 80% for manganese, and 93.3% for iron. For Ugandan standards, the samples exceeding the guideline values were 6.7% for magnesium, 13.3% for calcium, 13.3% for manganese, and 6.7% for iron. Therefore, the surface water from different sources is contaminated with major elements. The levels of metals in the surface water samples were higher than those in a study by Walakira and Okot-Okumu [39] which were reported as 17–39 mg/l for calcium, 0.6–53 mg/l for sodium, 0.05–0.26 mg/l for lead, 0.02–0.56 mg/l for copper, and below the detection level for cadmium. The main source of these elements could be weathering of mineralised soils and rocks as well as mobilisation during sorption-desorption processes for trace elements onto organic matter and soil particles.

**Figure 4:**
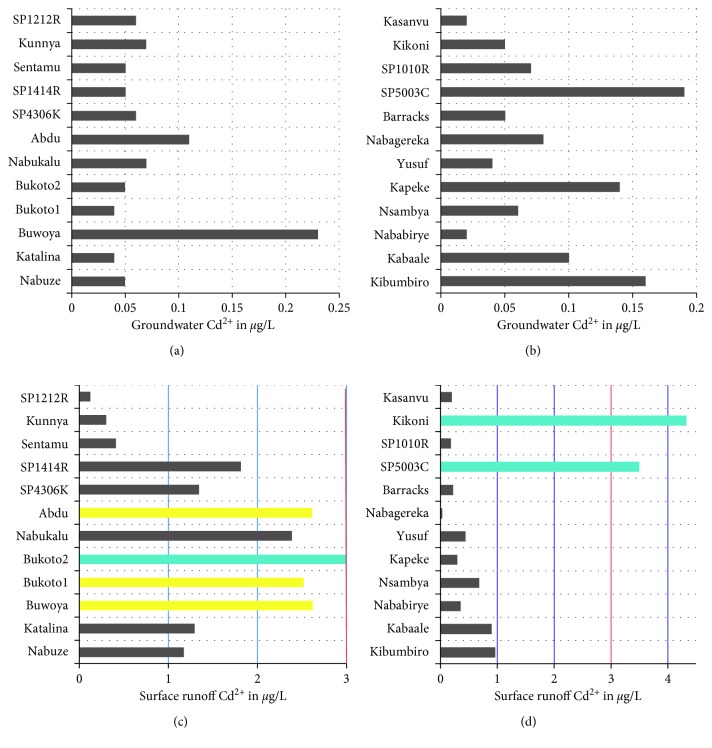
Outline of Cd(II) levels in the shallow groundwater and associated surface runoff from Kampala city (where the red line indicates the guideline value for levels of cadmium in surface water).

**Figure 5:**
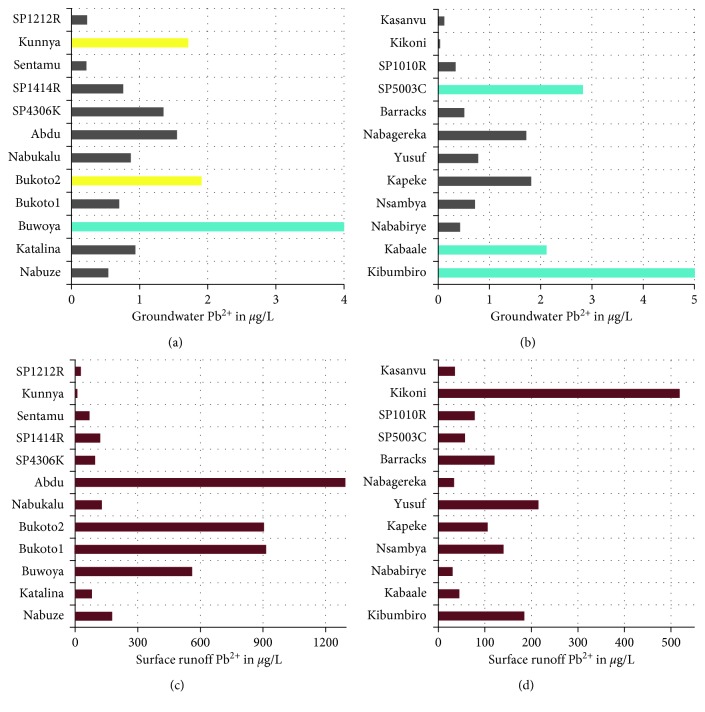
Outline of Pb(II) levels in the shallow groundwater and associated surface runoff from Kampala city (where the red bars indicate that the levels of lead in surface runoff were all the above guideline value).

Figures 4 and 5 give a synopsis of the levels of cadmium and lead in the shallow groundwater in comparison to the levels in surface runoff associated with the protected spring catchment areas.

The levels of Cd^2+^ ions in shallow groundwater samples were below allowable limits for groundwater, whereas the Cd^2+^ ions levels in surface runoff were 12% elevated and 12% above the limit of 3 *µ*g/L. For Pb^2+^ ions in shallow groundwater, 24% of the levels were elevated, whereas 100% of the levels of Pb^2+^ ions in surface runoff were by 3–130 times higher than the allowable limit of 10 *µ*g/L. The results in this study were higher than those for Lake Victoria water reported by Tole and Shitsama [40] which were 0.12–0.45 *µ*g/l for Pb and 0.01 *µ*g/l for Cd. Although the vertical mobility of the trace metals is controlled by the soil pH and organic matter content, the flood zones of the periurban areas where the shallow groundwater samples were picked tend to have mixing of surface water and shallow groundwater during the rainy seasons which may cause detrimental effects to the water quality of the shallow aquifers.

Tables 10 and 11 show the levels of trace elements in the surface water from various sample sites and the percentage of those samples above the guideline values, respectively. The trace elements levels exceeding the WHO guideline values in surface water were 33.3% for nickel, 25% for antimony, 31% for nickel, 6% for copper, 13% for zinc, 25% for arsenic, 6.7% for selenium, 13.4% for barium, and 40% for lead, whereas those exceeding the Ugandan guideline values were only 6.7% for copper although Taylor and Howard [32] reported minor exceedance above the WHO guideline values for barium, nickel, lead, uranium, and cadmium in their study of groundwater in Uganda. The urban streams and landfill leachate have elevated levels of trace metals: boron, antimony, arsenic, nickel, selenium, barium, and lead. The mine tailings are contaminated with copper and nickel. However, the pollution trends depict anthropogenic contamination although natural mobilisation of metal ions from the parent rock is possible. Nickel contamination results from the usage of electric cells and industrial wastewater, whereas copper was high in the copper mine tailings. Arsenic levels were evidently high in landfill leachates and Lubigi Channel which implied that anthropogenic pollution of the environment occurred since the levels in the shallow groundwater were all below the WHO guideline values.

Table 12 shows results of metal speciation studies for selected streams, waterways, and landfill leachates done by modelling the levels of elements using Visual MINTEQ. The results indicated that, in the landfill leachates, 74% of the metal ions are bound to dissolved organic matter (DOM) except for aluminium; 70% exists as inorganic aluminium, whereas most metal contaminants show toxic effects in their inorganic forms. The dominant ionic species in the landfill leachate are fulvic acid bound metals and metal hydroxides for nickel, copper, zinc, cadmium, lead, and iron, whereas manganese is a free ion and aluminium is in form of aluminium tetrahydroxyl radical. For the urban streams, 25% of the metal ions are bound to dissolved organic matter, 38% metal species are free metal ions, and 19% are in form of inorganic species.

The prominent ionic species in urban streams are manganese, nickel, zinc, and cadmium ions, iron(III) hydroxide, aluminium tetrahydroxyl radical, and fulvic acid bound copper and lead. The speciation of metal ions shows that they will be bound to DOM or fulvic acid; however, if the pH, DOC, and calcium content are lowered, the metal binding to the biotic ligand will increase causing toxicity effects to sensitive water plants and animals.

**Figure 6:**
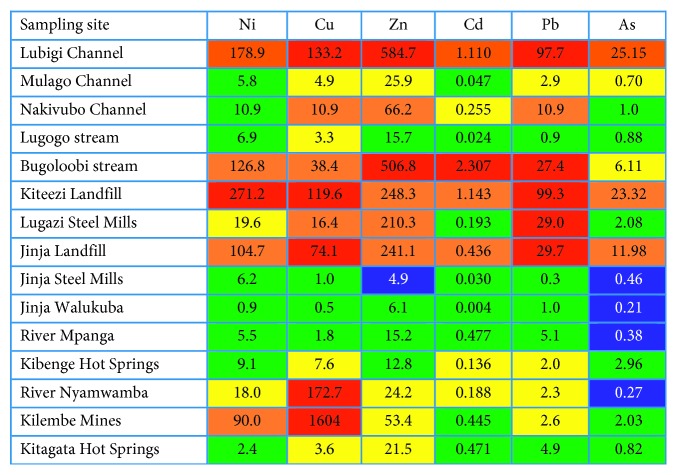
Comparison of concentrations of selected trace metal elements (*µ*g/L) in surface water from particular water sources in Lake Victoria basin, Uganda, with Swedish environmental guidelines.

**Figure 7:**
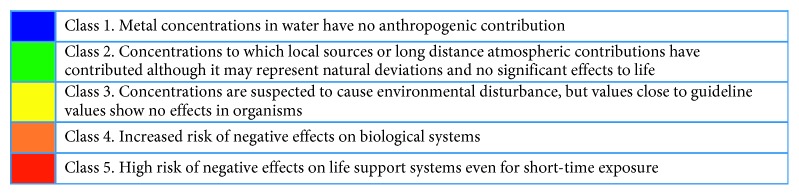
Colour codes used for the Swedish Environmental Protection Agency (Naturvårdsverket) criteria.

In Figure 6, a comparison of the trace metal elements in surface water with the Swedish environmental guidelines for lakes and watercourses is presented, whereas Table 13 showed the criteria used.  Figure 7 presents the description of the colour codes used in the criteria. The results showed that Lubigi Channel that traverses the periurban area of Kawempe Division and Bugoloobi Channel, whose catchment areas are in the industrial area of Kampala, has been shown to pose a high risk of negative effects on life support systems even for short-time exposure of exposure to Zn, Cu, Pb, and Cd metals. The leachate from Jinja and Kiteezi municipal landfills pose a high risk of exposure to Zn, Cu, Pb, and Cd metals. The effluent from Lugazi Steel Mills poses high risk of negative effects on life support systems even for short-time exposure to Pb, whereas the water from Kilembe Mine tailings and River Nyamwamba poses a greater risk of exposure to copper. The results from these case studies showed high risk of negative effects on life support systems even for short-time exposure. According to the results of this study, the water samples from Lugogo Channel in Kampala, Walukuba Channel in Jinja, River Mpanga in Kasese, and Kitagata Hot Springs presented metal concentrations in water that were suspected to have no anthropogenic contribution or trace metals contamination and no significant effects to life. Documented toxicity studies in surface water from Lake Victoria basin are scarce although in studies by Ljung [19] the trace metal levels that exceeded the lower limits for acute effects on organisms reported were 55% for cadmium, 5% for nickel, 77% for copper, 100% for zinc, and 33% for lead as compared to 46% for cadmium, 30% for nickel, 76% for copper, 46% for zinc, and 46% for lead for the current study.

The effects of selected trace metals on life support systems have been done basing on the criteria from the Swedish Environmental Protection Agency [29] and are shown in Figure 6. These results of the comparison and classification of predicted risks were more general for environmental health effects. The description of the criteria used in the classification of risks due to levels of metal ions in the aquatic environment is shown in Table 13. In the selected waterways, streams, and landfill leachate, there were suspected toxicity effects due to lead, copper, and zinc with Bugoloobi and Lubigi waterways being most risky. Due to the colour codes described in Figure 7, the levels of the metals in the aquatic environment that show no effects to moderate effects were accorded colours from yellow to blue. The values in blue such as those for arsenic are suspected to cause environmental disturbance, but values close to guideline values normally show no effects in organisms; moreover, the values in yellow are concentrations to which local sources or long distance atmospheric contributions have contributed although it may represent natural deviations and no significant effects to life.

The green coloured values are concentrations in water that have no anthropogenic contribution and are below guideline values. Therefore, continued discharge of untreated contaminated wastewater and landfill leachate will continue to impact on the quality of water in urban streams. The highest risk of exposure is due copper and lead followed by zinc and cadmium, whereas the water that poses the greatest risk of exposure is that from Lubigi Channel, Kiteezi Landfill leachate, Bugoloobi Channel, and Jinja Landfill leachate.

Tables 14 and 15 present results of modelling values of levels of elements in the surface water using the Bio-met software tool that is applied over a range of different organisms to calculate the 5^th^ percentile of the species sensitivity distribution (SSD).

HC5 aims at protecting at least 95% of the species and reflects the bioavailability conditions of a specific site according to the water chemistry. In Tables 14 and 15, the local HC5 values were in the ranges of 54.56–474.12 *µ*g/L for Cu^2+^, 29.95–151.90 *µ*g/L for Ni^2+^, 62.55–623.74 *µ*g/L for Zn^2+^, and 30.24–92.51 *µ*g/L for Pb^2+^ for selected surface water samples. The bioavailable metal concentration is the concentration of a metal that is bioavailable at the site or water body and were in the ranges of 0.02–29.40 *µ*g/L for Cu^2+^, 0.18–12.45 *µ*g/L for Ni^2+^, 0.96–74.19 *µ*g/L for Zn^2+^, and 0.03–1.50 *µ*g/L for Pb^2+^. The risk characterisation ratio (RCR) values that identified potential environmental risks for the selected surface water samples were 15.4% for Cu^2+^, 30.8% for Ni^2+^, 30.8% for Zn^2+^, and 15.4% for Pb^2+^. These results require more detailed risk assessments to inform policy on appropriate action.

The water source with the highest potential risk was Lubigi Channel. Johnson et al. [41] studied twelve metals in UK surface waters and found that the relative risk to organisms was highest for copper, aluminium, and zinc, whereas in this report, the highest risk of exposure to metals in surface waters was due to zinc and nickel.

Tables 16 and 17 present results of modelling values of levels of elements in the selected shallow groundwater sources using the Bio-met software tool that is applied over a range of different organisms to calculate the 5^th^ percentile of the species sensitivity distribution (SSD). The local HC5 values in Tables 16 and 17 were in the ranges of 4.15–8.55 *µ*g/L for Cu^2+^, 8.20–9.67 *µ*g/L for Ni^2+^, 10.90–14.81 *µ*g/L for Zn^2+^, and 2.70–5.38 *µ*g/L for Pb^2+^ for selected shallow groundwater samples. The bioavailable metal concentration values were in the ranges of 0.13–1.13 *µ*g/L for Cu^2+^, 1.03–12.20 *µ*g/L for Ni^2+^, 2.94–34.88 *µ*g/L for Zn^2+^, and 0.10–1.13 *µ*g/L for Pb^2+^. The risk characterisation ratio (RCR) values that identified potential environmental risks for the selected surface water samples were 4.1% for Cu^2+^, 45.8% for Ni^2+^, 62.5% for Zn^2+^, and 8.3% for Pb^2+^. These results require more detailed risk assessments to inform policy on appropriate action since the water sources are used for domestic purposes. The shallow groundwater source with the highest potential risk was Kibumbiro in Lubaga Division, whereas those that had RCR values below 1 required no action.

## 4. Conclusions

The shallow groundwater from protected springs in Kampala city had elevated levels of major and traces metals although some cases were below and others above the WHO guideline values, Ugandan standards, and Canadian standards. The levels of metals in surface water, landfill leachate, and surface runoff showed anthropogenic contamination and revealed increased risks of negative biological effects on organisms as well as the general environmental health. Speciation studies for selected streams, waterways, and landfill leachates using Visual MINTEQ indicated that 74% of the metal ions in the landfill leachates and 25% of the metal ions in urban streams are bound to DOM and were not available as free metal ions. Toxicity studies using the Bio-met software tool showed that selected surface water sources and shallow groundwater sources had potential environmental risks and require further detailed risk assessments to inform policy on appropriate action. However, the current state of the surface water quality indicates that the shallow groundwater in Kampala which is tapped from weathered regolith is acidic and vulnerable to contamination when episodes of acidic rain increase desorption of metal ions from organic matter and soil particles and thence the mobility of trace metals and contamination. The surface water, wastewater, landfill leachates, and surface runoff from several subcatchments of the Lake Victoria catchment zone are contaminated with trace and major elements most likely due to anthropogenic activities and natural mobilisation of metals through weathering of rocks. Otherwise continued discharge of untreated surface runoff, wastewater, and landfill leachate will negatively impact the shallow groundwater when the soil water is laden during the wet seasons, and the surface water interacts with the groundwater in shallow aquifers without reacting with the soil chemicals and thence groundwater contamination. There is need for intervention measures including legislature enforcement, monitoring, and alternative wastewater remediation action to curb water environment contamination.

## 5. Recommendations

To ensure guaranteed groundwater quality, the urban streams need be protected through treating the surface runoff, wastewater, and landfill leachate before discharge. The impending water disaster can be deterred with proactive measures such as building a time series data base on total water quality, regular monitoring of groundwater quality, and enforcing regulation preventive remediation. Further research will aim at modelling prediction of trace metal contaminants' mobility and distribution in the environment to be able to contaminate the meagre resource and avert environmental trace metal toxicity effects.

## Figures and Tables

**Table 1:** Coordinates of some of the sampling points that were plotted on the maps in Figures 1 and 2 using ArcGIS.

Sample site	Latitude (N)			Longitude (E)		
Units	° (degrees)	′ (minutes)	″ (seconds)	° (degrees)	′ (minutes)	″ (seconds)
Katarina	00	20	58	32	37	48
Nabuze	00	20	38	32	38	28
Buwooya	00	21	30	32	38	0
Bukoto 1	00	21	29	32	36	0
Bukoto 2	00	21	10	32	35	3
Nabukalu	00	21	30	32	33	4
Abdu	00	21	0	32	33	0
SP4306K	00	20	5	32	33	0
Kikoni	00	19	53	32	33	4
SP1414R	00	20	44	32	32	0
Sentamu	00	20	40	32	32	0
Kunnya	00	20	10	32	32	0
SP5003C	00	19	26	32	34	0
SP1212R	00	20	0	32	32	0
Kibumbiro	00	19	10	32	31	2
Kabaale	00	18	40	32	31	0
Nababirye	00	19	36	32	32	10
SP1010R	00	20	37	32	33	24
Kapeke	00	17	47	32	34	5
Yusuf	00	18	10	32	34	5
Kasanvu	00	18	25	32	36	45
Nsambya	00	18	29	32	36	45
Nabagereka	00	18	40	32	34	9
Barracks	00	19	11	32	34	10
Lubigi	00	21	01	32	33	56
Mulago	00	20	44	32	34	11
Nakivubo	00	18	56	32	35	43
Lugogo	00	19	19	32	36	34
Bugoloobi	00	19	07	32	37	0
Kiteezi	00	24	49	32	34	37

**Table 2:** Major elements (mg/L) in water from selected protected springs in Kampala city (*n*=3, ±standard deviation) compared with WHO guideline values and the Ugandan standards [28, 30].

WHO guideline values	Na	Mg	Al	K	Ca	Cr	Mn	Fe
	200	100	0.2	10	200	0.05	0.4	0.3
Ugandan standards	200	50	0.1	—	75	0.05	0.1	0.3
Sample site								
*Nakawa division*								
Nabuze	23 ± 0.3	4.7 ± 1.2	0.002	3.6 ± 0.4	11.6 ± 1.3	0.051 ± 0.004	0.14 ± 0.01	0.3 ± 0.01
Katarina	14 ± 0.6	2.2 ± 0.7	0.002	4.4 ± 0.5	6.7 ± 0.8	0.020 ± 0.006	0.02 ± 0.01	0.2 ± 0.01
Buwooya	10 ± 1.5	1.8 ± 0.2	0.191	3.6 ± 0.3	5.7 ± 1.5	0.021 ± 0.003	0.02 ± 0.01	0.7 ± 0.03
Bukoto 1	10 ± 1.2	1.3 ± 0.3	0.098	2.6 ± 0.2	4.9 ± 1.0	0.012 ± 0.007	0.06 ± 0.02	0.5 ± 0.02
Bukoto 2	23 ± 1.2	2.0 ± 0.4	0.360	5.7 ± 0.5	9.2 ± 1.3	0.011 ± 0.004	0.24 ± 0.01	0.7 ± 0.02

*Kawempe division*								
Nabukalu	37 ± 1.9	4.1 ± 0.4	0.012	8.3 ± 0.5	13.5 ± 1.0	0.007 ± 0.006	0.33 ± 0.01	0.3 ± 0.02
Abdu	61 ± 2.3	6.5 ± 0.6	0.167	17.2 ± 1.0	21.4 ± 2.1	0.007 ± 0.005	1.14 ± 0.02	0.4 ± 0.01
SP4306K	40 ± 3.0	4.4 ± 0.8	0.001	15.1 ± 1.3	17.1 ± 1.2	0.007 ± 0.007	0.84 ± 0.02	0.3 ± 0.02
Kikoni	20 ± 2.9	3.1 ± 0.7	0.001	4.9 ± 0.6	11.1 ± 0.6	0.006 ± 0.001	0.62 ± 0.03	0.2 ± 0.01

*Rubaga division*								
SP1414R	31 ± 2.5	4.1 ± 0.2	0.001	6.5 ± 0.5	12.0 ± 1.3	0.008 ± 0.002	0.24 ± 0.02	0.2 ± 0.01
Sentamu	27 ± 2.6	3.8 ± 0.1	0.001	7.9 ± 0.6	13.0 ± 1.1	0.007 ± 0.001	0.24 ± 0.01	0.2 ± 0.01
Kunnya	23 ± 2.2	1.9 ± 0.1	0.107	8.6 ± 0.3	8.3 ± 0.4	0.006 ± 0.002	0.28 ± 0.04	0.4 ± 0.02
SP1212R	22 ± 1.7	3.7 ± 0.2	0.001	8.3 ± 0.5	11.0 ± 0.5	0.005 ± 0.002	0.14 ± 0.02	0.1 ± 0.03
Kibumbiro	23 ± 1.8	2.7 ± 0.3	0.299	5.7 ± 0.4	11.2 ± 0.3	0.006 ± 0.001	0.78 ± 0.02	0.7 ± 0.02
Kabaale	19 ± 1.3	3.2 ± 0.4	0.169	7.6 ± 0.2	14.3 ± 0.3	0.007 ± 0.003	0.09 ± 0.01	0.5 ± 0.03
Nababirye	8.0 ± 0.2	2.4 ± 0.2	0.002	4.2 ± 0.1	7.1 ± 0.1	0.010 ± 0.002	0.02 ± 0.01	1.9 ± 0.05
SP1010R	35 ± 1.9	4.2 ± 0.3	0.002	9.7 ± 0.4	12.7 ± 0.4	0.005 ± 0.001	0.53 ± 0.02	0.2 ± 0.03

*Makindye division*								
Nsambya	45.8 ± 2.3	4.4 ± 0.2	0.002	11.1 ± 0.6	14.7 ± 0.3	0.005 ± 0.003	0.58 ± 0.01	0.2 ± 0.02
Kapeke	66.4 ± 3.3	4.9 ± 0.1	0.015	12.2 ± 0.2	17.2 ± 0.2	0.005 ± 0.002	1.20 ± 0.02	0.2 ± 0.05
Yusuf	23.2 ± 1.1	2.8 ± 0.1	0.001	7.4 ± 0.5	11.7 ± 0.1	0.005 ± 0.001	0.09 ± 0.01	0.4 ± 0.06
Kasanvu	21.0 ± 1.3	4.0 ± 0.2	0.001	5.2 ± 0.1	18.7 ± 0.3	0.005 ± 0.002	0.02 ± 0.00	0.2 ± 0.02

*Central division*								
Nabagereka	43.7 ± 2.3	5.3 ± 0.2	0.150	15.1 ± 0.4	23.5 ± 0.1	0.006 ± 0.002	0.74 ± 0.01	0.5 ± 0.02
Barracks	25.1 ± 1.2	4.6 ± 0.3	0.001	5.9 ± 0.1	20.2 ± 0.1	0.006 ± 0.001	0.04 ± 0.00	0.2 ± 0.01
SP5003C	37.5 ± 2.4	5.1 ± 0.1	0.101	14.9 ± 0.3	17.3 ± 0.2	0.007 ± 0.001	0.27 ± 0.01	0.4 ± 0.03

**Table 3:** A summary of the percentage of selected protected springs in Kampala city with levels of major elements in water above and below the WHO guideline values and the Ugandan standards.

Element	Na	Mg	Al	K	Ca	Cr	Mn	Fe
Springs above Ugandan guidelines	0	0	20	—	0	4	66.7	58.3
Springs below Ugandan guidelines	100	100	80	—	100	96	33.3	41.7
Springs above WHO guidelines	0	0	8	33.3	0	4	29	58.3
Springs below WHO guidelines	100	100	92	67.7	100	96	71	41.7

**Table 4:** Levels of trace elements (*µ*g/L) in water from selected protected springs in Kampala city in comparison to WHO guideline values [30, 33, 34].

	B	Sb	Ni	Cu	Zn	Cd	Ba	Pb
WHO guidelines	2400	20	70	2000	3000	3	700	10
Ugandan standards	—	—	—	1000	5000	10	1000	50
Canadian guidelines	5000	6	—	1000	5000	5	1000	10
Sample site								
*Nakawa division*								
Nabuze	2.0 ± 0.1	1.44 ± 0.03	9.8 ± 0.3	1.4 ± 0.1	9.4 ± 0.5	0.05 ± 0.002	150 ± 5.5	0.54 ± 0.01
Katarina	2.3 ± 0.4	0.45 ± 0.01	6.8 ± 0.4	3.2 ± 0.2	16.9 ± 0.8	0.04 ± 0.001	146 ± 4.3	0.94 ± 0.02
Buwooya	2.1 ± 0.9	0.38 ± 0.01	3.9 ± 0.6	1.3 ± 0.1	16.0 ± 0.4	0.23 ± 0.011	73 ± 3.4	4.09 ± 0.09
Bukoto 1	2.1 ± 0.5	0.42 ± 0.02	3.2 ± 0.2	0.8 ± 0.2	13.6 ± 0.1	0.04 ± 0.001	140 ± 5.6	0.70 ± 0.01
Bukoto 2	2.5 ± 0.1	0.39 ± 0.02	5.4 ± 0.3	1.8 ± 0.3	26.4 ± 0.4	0.05 ± 0.002	234 ± 7.7	1.91 ± 0.02

*Kawempe division*								
Nabukalu	2.4 ± 0.1	0.40 ± 0.01	7.4 ± 0.1	1.6 ± 0.2	20.9 ± 0.4	0.07 ± 0.01	332 ± 4.5	0.87 ± 0.03
Abdu	2.7 ± 0.2	0.55 ± 0.02	12 ± 0.1	2.0 ± 0.1	22.9 ± 0.6	0.11 ± 0.02	821 ± 8.3	1.55 ± 0.02
SP4306K	2.7 ± 0.2	10.98 ± 0.02	8.6 ± 0.1	2.0 ± 0.2	14.7 ± 0.1	0.06 ± 0.01	422 ± 7.2	1.35 ± 0.01
Kikoni	3.2 ± 0.3	0.21 ± 0.01	3.8 ± 0.3	0.5 ± 0.1	6.9 ± 0.2	0.05 ± 0.01	180 ± 6.3	0.04 ± 0.01

*Rubaga division*								
SP1414R	2.0 ± 0.4	9.19 ± 0.01	9.1 ± 0.2	1.7 ± 0.5	17.1 ± 0.7	0.05 ± 0.01	397 ± 5.1	0.76 ± 0.01
Sentamu	1.7 ± 0.5	0.41 ± 0.02	7.4 ± 0.1	0.6 ± 0.3	9.3 ± 0.0	0.05 ± 0.01	303 ± 7.7	0.22 ± 0.03
Kunnya	2.4 ± 0.1	0.90 ± 0.01	6.1 ± 0.7	1.8 ± 0.4	24.5 ± 0.7	0.07 ± 0.02	249 ± 6.5	1.71 ± 0.02
SP1212R	1.7 ± 0.0	0.86 ± 0.02	8.5 ± 0.4	0.7 ± 0.3	9.8 ± 0.0	0.06 ± 0.02	246 ± 8.0	0.23 ± 0.01
Kibumbiro	3.9 ± 0.1	1.34 ± 0.04	15.3 ± 0.0	3.7 ± 0.5	48.0 ± 0.5	0.16 ± 0.03	454 ± 7.9	5.06 ± 0.05
Kabaale	5.1 ± 0.5	0.90 ± 0.03	4.9 ± 0.1	1.7 ± 0.8	16.5 ± 0.6	0.10 ± 0.02	286 ± 8.3	2.11 ± 002
Nababirye	1.9 ± 0.6	1.80 ± 0.00	4.9 ± 0.2	5.5 ± 0.8	7.7 ± 0.8	0.02 ± 0.01	108 ± 3.1	0.43 ± 0.03
SP1010R	2.2 ± 0.5	0.22 ± 0.01	8.1 ± 0.2	0.7 ± 0.7	12.1 ± 0.1	0.07 ± 0.02	442 ± 5.8	0.34 ± 0.01

*Makindye division*								
Nsambya	2.5 ± 0.2	1.75 ± 0.02	15.2 ± 0.4	1.4 ± 0.6	11.7 ± 0.6	0.06 ± 0.01	506 ± 6.3	0.72 ± 0.02
Kapeke	2.8 ± 0.7	4.15 ± 0.03	25.5 ± 0.8	3.4 ± 0.8	38.0 ± 0.4	0.14 ± 0.02	1132 ± 8.9	1.81 ± 0.04
Yusuf	2.6 ± 0.1	0.64 ± 0.01	2.1 ± 0.1	0.9 ± 0.5	11.5 ± 0.3	0.04 ± 0.01	118 ± 5.3	0.78 ± 0.05
Kasanvu	7.2 ± 0.8	0.44 ± 0.01	3.5 ± 0.0	0.9 ± 0.2	4.1 ± 0.2	0.02 ± 0.00	117 ± 6.9	0.12 ± 0.04

*Central division*								
Nabagereka	3.5 ± 0.2	0.38 ± 0.02	8.94 ± 0.4	1.5 ± 0.9	15.9 ± 1.3	0.08 ± 0.01	508 ± 9.0	1.72 ± 0.02
Barracks	3.0 ± 0.8	5.74 ± 0.08	8.22 ± 0.3	1.1 ± 0.8	7.6 ± 0.7	0.05 ± 0.01	213 ± 5.1	0.51 ± 0.01
SP5003C	2.3 ± 0.2	0.52 ± 0.05	4.52 ± 0.1	1.4 ± 0.4	25.1 ± 1.5	0.19 ± 0.02	424 ± 6.4	2.82 ± 0.01

**Table 5:** A summary of the percentage of selected protected springs in Kampala city with levels of trace elements in water above and below the WHO guidelines, Canadian guidelines, and Ugandan standards.

Element	B	Sb	Ni	Cu	Zn	Cd	Ba	Pb
Springs above Ugandan guidelines	—	—	—	0	0	0	0	0
Springs below Ugandan guidelines	—	—	—	100	100	100	100	100
Springs above WHO guidelines	0	0	0	0	0	0	8.3	0
Springs below WHO guidelines	100	100	100	100	100	100	91.7	100
Springs above Canadian guidelines	0	8.3	—	0	0	0	0	0
Springs below Canadian guidelines	100	91.7	—	100	100	100	100	100

**Table 6:** Mean values of other parameters of shallow groundwater and associated surface runoff from periurban Kampala compared to Guidelines for Canadian Drinking Water Quality and Ugandan Standards.

Spring name	Analysis of spring water							Surface runoff analysis		
	T (°C)	pH	DOC (mg/L)	EC (*µ*S/cm)	NO_3_^−^ (mg/L)	Cl^−^ (mg/L)	F^−^ (mg/L)	EC (*µ*S/cm)	Cd^2+^ (*µ*g/L)	Pb^2+^ (*µ*g/L)
Canadian guidelines	—	6.5–8.5	—	—	45	—	0.5	—	5	10
Ugandan standards	—	6.0–8.5	—	1000	10	250	1.0	1000	10	50
*Nakawa division*										
Nabuze	25.0	5.46	1.0	665	34.1	11.9	0.1	750	1.18	178
Katarina	24.0	5.36	1.0	372	19.9	6.9	0.1	450	1.30	81
Buwooya	25.0	5.45	1.0	211	10.4	3.8	0.2	350	2.62	560
Bukoto 1	26.0	5.07	1.0	226	27.0	13.3	0.3	300	2.52	915
Bukoto 2	25.0	4.80	2.0	470	64.3	24.2	0.2	520	3.10	905
Kiddumu	25.0	5.72	1.0	862	44.8	16.8	0.1	1208	0.32	108

*Kawempe division*										
Nabukalu	25.0	4.93	2.0	873	78.7	30.3	0.1	980	2.39	129
Abdu	26.0	4.74	1.0	2650	78.5	46.5	0.1	3050	2.64	1294
SP4306K	25.0	4.98	1.0	786	38.6	16.6	0.1	824	1.35	95.8
Kikoni	26.0	5.35	1.0	497	42.5	18.2	0.1	532	8.00	519
Bukuku	26.0	5.86	2.0	643	65.4	31.2	0.1	835	1.19	92.1

*Rubaga division*										
SP1414R	26.0	4.93	1.0	774	63.6	27.0	0.1	812	1.82	121
Sentamu	25.0	4.98	1.0	722	74.6	32.3	0.1	820	0.42	69
Kunnya	25.0	5.30	1.0	574	107.3	41.7	0.1	642	0.31	11
SP1212R	25.0	5.03	1.0	631	1.0	10.2	1.0	722	0.13	28
Kibumbiro	24.0	5.14	2.0	586	68.4	40.1	0.1	650	0.96	185
Kabaale	25.0	5.32	1.0	607	70.2	28.4	0.2	600	0.90	45
Nababirye	25.0	5.68	1.0	272	42.5	18.3	0.1	472	0.35	31
Nsereko	26.0	4.89	1.0	786	24.7	8.8	0.1	786	24.7	8.8
Lugala	25.0	5.18	1.0	272	57.9	21.6	0.1	1090	0.30	30.8
SP1010R	25.0	5.18	1.0	916	64.5	28.4	0.1	1200	0.03	78

*Makindye division*										
Nsambya	25.0	5.37	1.0	1098	101.2	42.1	0.2	1250	0.68	140
Kapeke	25.0	5.18	1.0	1472	102.5	64.4	0.2	1670	0.29	106
Yusuf	25.0	5.45	1.0	648	101.4	42.7	0.2	1210	0.43	215
Kasanvu	25.0	5.68	1.0	764	38.7	11.8	0.5	825	0.18	36
Kibuuka	25.0	5.89	1.0	873	45.6	18.6	0.2	972	1.20	201.4
Kisugu	25.0	5.68	1.0	764	125.3	58.5	0.2	1250	0.68	140.0
SP7009M	26.0	5.24	2.0	607	45.6	18.6	0.2	648	0.82	141.0

*Central division*										
Nabagereka	25.0	5.54	2.0	1208	58.3	35.6	0.3	779	0.03	34
Barracks	25.0	5.36	1.0	779	74.7	23.2	0.1	864	0.22	121
SP5003C	24.0	5.55	2.0	1051	53.7	35.5	0.3	1251	0.29	57
Kisekka	26.0	4.97	2.0	772	5.1	49.6	5.0	820	18.3	15.4
Kinyoro	24.0	5.20	1.0	421	18.0	5.9	0.1	774	1.05	294.9
Bativa	26.0	4.95	1.0	832	38.8	14.8	0.1	870	0.68	55.7
SP5508C	25.0	5.24	1.0	680	44.4	29.4	0.2	680	44.4	29.4
SP5303C	26.0	5.25	2.0	648	53.7	36.5	0.3	648	53.7	36.5
SP4902C	26.0	4.92	1.0	786	60.5	23.2	0.1	1670	0.29	105.8

**Table 7:** A summary of the percentage of samples of shallow groundwater and associated surface runoff from periurban Kampala above and below the Guidelines for Canadian Drinking Water Quality and Ugandan Standards.

Sample site	Protected spring water			Surface runoff		
Parameter	EC	NO_3_^−^	Cl^−^	EC	Cd^2+^	Pb^2+^
Springs above Ugandan guidelines	13.5	94.6	—	21.6	2.7	81.1
Springs below Ugandan guidelines	86.5	5.4	—	78.4	97.3	18.9
Springs above Canadian guidelines	—	73	0	—	5.4	97.3
Springs below Canadian guidelines	—	27	100	—	94.6	2.7

**Table 8:** Mean levels of major elements (mg/L) and other parameters of surface water from selected streams and waterways in Lake Victoria basin, Uganda, compared to the WHO guidelines and Ugandan standards (maximum concentrations).

Sampling site	WHO water guidelines									
	pH	EC (*µ*S/cm)	T (C°)	Na	Mg	Al	K	Ca	Mn	Fe
	6.5–8.5	1000	25	200	50	0.1	10	200	0.1	0.3
	Ugandan standards									
	6.0–8.0	1000	23–25	—	100	0.5	—	100	1.0	10
Lubigi Channel	7.49	1530	29	51.5	5.1	0.066	25.03	26.3	0.80	2.61
Mulago Channel	7.02	1420	25	50.7	4.6	0.062	22.60	25.5	0.83	2.63
Nakivubo Channel	7.20	1723	28	56.4	5.4	0.154	28.62	24.3	0.53	2.84
Lugogo stream	7.16	930	26	31.8	5.7	0.001	17.32	26.3	0.83	3.49
Bugoloobi stream	7.09	700	25	19.9	5.1	0.002	6.28	18.0	0.13	0.33
Kiteezi Landfill	8.44	5100	30	5245	82.8	0.140	0.03	26.2	0.29	5.35
Lugazi Steel Mills	7.68	133	25	40.7	4.6	0.739	27.06	27.5	0.62	14.98
Jinja Landfill	7.89	3120	27	400	184.8	0.002	0.07	135.4	2.87	6.49
Jinja Steel Mills	7.46	1772	27	36.1	22.5	0.001	4.66	62.6	0.68	1.14
Jinja Walukuba	7.90	300	26	11.9	1.7	0.001	3.49	5.8	0.01	0.20
River Mpanga	6.80	768	25	27.8	15.2	0.001	6.84	82.7	1.57	1.27
Kibenge Hot Springs	7.60	924	46	600.2	4.4	0.040	19.99	199	0.03	0.97
River Nyamwamba	7.03	127	22	6.4	3.8	0.002	3.62	10.0	0.13	1.21
Kilembe Mines	7.45	1569	26	509.8	25.1	0.924	518.10	78.6	0.45	0.93
Kitagata Hot Springs	7.80	1182	59	205.6	0.2	0.001	10.50	30.5	0.02	0.33

**Table 9:** A summary of the percentage of samples of surface water from selected streams and waterways in Lake Victoria basin, Uganda, with mean levels of major elements and other parameters of surface water from selected streams and waterways in Lake Victoria basin, Uganda, above and below the WHO guidelines and Ugandan standards.

Element	EC	Na	Mg	Al	K	Ca	Mn	Fe
Springs above Ugandan guidelines	53.5	—	6.7	26.7	—	13.3	13.3	6.7
Springs below Ugandan guidelines	46.7	—	93.3	73.3	—	86.7	86.7	93.3
Springs above WHO guidelines	53.5	33.3	13.3	33.3	53.5	0	80	93.3
Springs below WHO guidelines	46.7	66.7	86.7	66.7	46.7	100	20	6.7

**Table 10:** Trace metal elements (*µ*g/L) in surface water from selected streams and waterways in Lake Victoria basin, Uganda, compared with WHO guidelines [30] and Ugandan standards [28, 37].

Sampling site	WHO water guidelines								
	B	Ni	Cu	Zn	As	Se	Cd	Ba	Pb
	2400	70	2000	4000	10	40	3	700	10
	Ugandan standards								
	5000	1000	1000	5000	200	1000	100	10000	100
Lubigi stream	485	178.9	133.2	584.7	25.15	27.71	1.110	6227	97.7
Mulago stream	14	5.8	4.9	25.9	0.70	0.88	0.047	215.1	2.9
Nakivubo stream	32	10.9	10.9	66.2	1.0	1.19	0.255	181.9	10.9
Lugogo stream	18	6.9	3.3	15.7	0.88	1.02	0.024	218.7	0.9
Bugoloobi stream	199	126.8	38.4	506.8	6.11	16.46	2.307	2723.7	27.4
Kiteezi Landfill leachate	1338	271.2	119.6	248.3	23.32	71.37	1.143	214.5	99.3
Lugazi Steel Mills effluent	126	19.6	16.4	210.3	2.08	0.69	0.193	73.3	29.0
Jinja Landfill leachate	953	104.7	74.1	241.1	11.98	25.62	0.436	186.3	29.7
Jinja Steel Mills effluent	29	6.2	1.0	4.9	0.46	2.55	0.030	104.8	0.3
Jinja Walukuba effluent	10	0.9	0.5	6.1	0.21	0.21	0.004	30.2	1.0
River Mpanga	11	5.5	1.8	15.2	0.38	0.47	0.477	341.7	5.1
Kibenge Hot Springs	296	9.1	7.6	12.8	2.96	23.18	0.136	25.8	2.0
River Nyamwamba	7.5	18.0	172.7	24.2	0.27	0.29	0.188	28.6	2.3
Kilembe Mines effluent	6.8	90.0	1604	53.4	2.03	0.60	0.445	31.0	2.6
Kitagata Hot Springs	375	2.4	3.6	21.5	0.82	2.60	0.471	36.8	4.9

**Table 11:** A summary of the percentage of samples of surface water from selected streams and waterways in Lake Victoria basin, Uganda, with mean levels of trace elements above and below the WHO guidelines and Ugandan standards.

Element	B	Ni	Cu	Zn	As	Cd	Ba	Pb
Springs above Ugandan guidelines	0	0	6.7	0	0	0	0	0
Springs below Ugandan guidelines	100	100	93.3	100	100	100	100	100
Springs above WHO guidelines	0	33.3	0	0	0	0	13.4	40
Springs below WHO guidelines	100	66.7	100	100	100	100	86.6	60

**Table 12:** Percentage composition of metal ions in aqueous media as determined by speciation studies of selected surface water samples from streams in Lake Victoria basin, Uganda.

Sampling site	Ni^2+^	Cu^2+^	Zn^2+^	Cd^2+^	Pb^2+^	Al^3+^	Mn^2+^	Fe^3+^
*Jinja Landfill leachate*								
Bound to DOM	92.5	100	96.3	87.7	99.9	46.9	24.5	100
Free ion	7.0	—	2.9	5.2	—	—	65.5	—
Inorganic	0.5	—	0.8	7.1	0.01	54.1	10.0	—
Dominant ion	FANi^+^	FA_2_CuOH	FA_2_Zn	FA_2_Cd	FA_2_Pb	Al(OH)_4_^−^	Mn^2+^	FA_2_FeOH

*Kiteezi Landfill leachate*								
Bound to DOM	96.5	100	99.4	97.5	100	13.5	37.1	100
Free ion	3.4	—	0.4	0.9	—	—	61.9	—
Inorganic	0.1	—	0.2	1.8	—	86.5	1.0	—
Dominant ion	FANi^+^	FA_2_CuOH	FA_2_Zn	FA_2_Cd	FA_2_Pb	Al(OH)_4_^−^	Mn^2+^	FA_2_FeOH

*Lubigi Channel*								
Bound to DOM	58.5	98.3	35.1	31.8	97.7	4.7	8.2	97.5
Free ion	40.0	0.7	59.8	58.4	1.6	—	86.3	—
Inorganic	1.5	1.0	6.1	10.8	1.3	95.3	5.5	2.4
Dominant ion	Ni^2+^	FA_2_Cu	Zn^2+^	Cd^2+^	FA_2_Pb	Al(OH)_4_^−^	Mn^2+^	FA_2_FeOH

*Nakivubo Channel*								
Bound to DOM	9.2	47.1	3.2	2.8	42.6	0.5	0.9	45.7
Free ion	86.9	27.6	87.9	76.6	38.7	—	90.3	—
Inorganic	3.9	25.3	8.9	20.6	18.7	99.5	8.8	54.3
Dominant ion	Ni^2+^	FA_2_Cu	Zn^2+^	Cd^2+^	FA_2_Pb	Al(OH)_4_^−^	Mn^2+^	Fe(OH)_3_

**Table 13:** The Swedish Environmental Protection Agency (Naturvårdsverket) criteria.

Metal (*µ*g/l)	Class 1 (very low)	Class 2 (low)	Class 3 (moderate)	Class 4 (high)	Class 5 (very high)
Cd	<0.01	0.01–0.1	0.1–0.3	0.3–1.5	>1.5
Cu	<0.5	0.5–3	3–9	9–45	>45
Ni	<0.7	0.7–15	15–45	45–225	>225
Pb	<0.2	0.2–1	1–3	3–15	>15
Zn	<5	5–20	20–60	60–300	>300

**Table 14:** Results for risk assessment with respect to zinc and lead for selected surface water samples.

Sample name	Local HC5 (dissolved) (*µ*g/L)	BioF	Bioavailable zinc concentration (*µ*g/L)	RCR	Local HC5 (dissolved) (*µ*g/L)	BioF	Bioavailable lead concentration (*µ*g/L)	RCR
River Mpanga	75.47	0.14	2.05	0.19	65.88	0.02	0.09	0.08
Nakivubo Channel	149.16	0.07	4.76	0.44	75.30	0.02	0.17	0.14
Lubigi Channel	183.54	0.06	34.68	**3.18**	78.08	0.02	1.50	**1.25**
Kibenge Hot Springs	105.39	0.10	1.22	0.11	68.61	0.02	0.03	0.03
River Nyamwamba	66.13	0.16	3.82	0.35	65.57	0.02	0.04	0.04
Kitagata Hot Springs	233.22	0.05	0.96	0.09	86.05	0.01	0.07	0.06
Kilembe Mine tailings	84.19	0.13	6.73	0.62	30.24	0.04	0.10	0.09
Mulago Channel	62.55	0.17	4.34	0.40	40.48	0.03	0.09	0.07
Bugoloobi Channel	74.31	0.15	74.19	**6.81**	66.34	0.02	0.50	0.41
Lugogo Channel	75.77	0.14	2.11	0.19	40.80	0.03	0.03	0.02
Lugazi Channel	150.28	0.07	15.16	**1.39**	67.58	0.02	0.51	0.43
Jinja Landfill leachate	111.33	0.10	23.50	**2.16**	77.56	0.02	0.46	0.38
Kiteezi Landfill leachate	623.74	0.02	4.32	0.40	92.51	0.01	1.29	**1.07**

**Table 15:** Results for risk assessment with respect to copper and nickel for selected surface water samples.

Sample name	Local HC5 (dissolved) (*µ*g/L)	BioF	Bioavailable copper concentration (*µ*g/L)	RCR	Local HC5 (dissolved) (*µ*g/L)	BioF	Bioavailable nickel concentration (*µ*g/L)	RCR
River Mpanga	119.21	0.01	0.02	0.02	53.20	0.08	0.41	0.10
Nakivubo Channel	185.25	0.01	0.06	0.06	59.21	0.07	0.74	0.18
Lubigi Channel	191.68	0.01	0.69	0.69	57.50	0.07	12.45	**3.11**
Kibenge Hot Springs	227.55	0.00	0.03	0.03	56.31	0.07	0.65	0.16
River Nyamwamba	131.48	0.01	1.32	**1.32**	42.11	0.09	1.71	0.43
Kitagata Hot Springs	159.16	0.01	0.02	0.02	53.90	0.07	0.18	0.04
Kilembe Mine tailings	54.56	0.02	29.40	**29.40**	29.95	0.13	12.02	**3.00**
Mulago Channel	75.42	0.01	0.06	0.06	30.80	0.13	0.75	0.19
Bugoloobi Channel	144.60	0.01	0.27	0.27	43.10	0.09	11.79	**2.95**
Lugogo Channel	82.05	0.01	0.04	0.04	31.76	0.13	0.87	0.22
Lugazi Channel	117.74	0.01	0.14	0.14	39.35	0.10	1.99	0.50
Jinja Landfill leachate	474.12	0.00	0.16	0.16	151.90	0.03	2.77	0.69
Kiteezi Landfill leachate	173.20	0.01	0.69	0.69	100.41	0.04	10.80	**2.70**

**Table 16:** Results for risk assessment with respect to copper and nickel for selected shallow groundwater samples.

Sample name	Local HC5 (dissolved) (*µ*g/L)	BioF	Bioavailable copper concentration (*µ*g/L)	RCR	Local HC5 (dissolved) (*µ*g/L)	BioF	Bioavailable nickel concentration (*µ*g/L)	RCR
Nabuze	4.95	0.20	0.28	0.28	8.20	0.49	4.78	**1.20**
Katarina	4.95	0.20	0.64	0.64	8.20	0.49	3.34	0.83
Buwooya	4.95	0.20	0.26	0.26	8.20	0.49	1.93	0.48
Bukoto 1	4.95	0.20	0.17	0.17	8.20	0.49	1.57	0.39
Bukoto 2	7.10	0.14	0.26	0.26	9.67	0.41	2.25	0.56
Nabukalu	7.10	0.14	0.22	0.22	9.67	0.41	3.05	0.76
Abdu	4.15	0.24	0.48	0.48	8.20	0.49	5.91	**1.48**
SP4306K	4.95	0.20	0.40	0.40	8.20	0.49	4.20	**1.05**
Kikoni	4.95	0.20	0.09	0.09	8.20	0.49	1.87	0.47
SP1414R	4.95	0.20	0.33	0.33	8.20	0.49	4.45	**1.11**
Sentamu	4.95	0.20	0.13	0.13	8.20	0.49	3.62	0.90
Kunya	4.95	0.20	0.37	0.37	8.20	0.49	3.01	0.75
SP1212R	4.95	0.20	0.15	0.15	8.20	0.49	4.17	**1.04**
Kibumbiro	7.10	0.14	0.53	0.53	9.67	0.41	6.33	**1.58**
Kabaale	4.95	0.20	0.36	0.36	8.20	0.49	2.40	0.60
Nababirye	4.95	0.20	**1.13**	1.13	8.20	0.49	2.40	0.60
SP1010R	4.95	0.20	0.16	0.16	8.20	0.49	3.96	**0.99**
Nsambya	4.95	0.20	0.29	0.29	8.20	0.49	7.42	**1.85**
Kapeke	4.95	0.20	0.70	0.70	8.20	0.49	12.20	**3.05**
Yusuf	4.95	0.20	0.19	0.19	8.20	0.49	1.03	0.26
Kasanvu	4.95	0.20	0.19	0.19	8.20	0.49	1.71	0.43
Nabagereka	8.55	0.12	0.17	0.17	9.67	0.41	3.70	**0.92**
Barracks	4.15	0.24	0.26	0.26	8.20	0.49	4.01	**1.00**
SP5003C	7.10	0.14	0.20	0.20	9.67	0.41	1.87	0.47

**Table 17:** Results for risk assessment with respect to zinc and lead for selected shallow groundwater samples.

Sample name	Local HC5 (dissolved) (*µ*g/L)	BioF	Bioavailable zinc concentration (*µ*g/L)	RCR	Local HC5 (dissolved) (*µ*g/L)	BioF	Bioavailable lead concentration (*µ*g/L)	RCR
Nabuze	11.56	0.94	7.93	0.73	2.70	0.44	0.24	0.20
Katarina	10.90	1.00	15.90	**1.46**	2.70	0.44	0.42	0.35
Buwooya	10.90	1.00	15.00	**1.38**	2.70	0.44	1.82	**1.51**
Bukoto 1	10.90	1.00	12.60	**1.16**	2.70	0.44	0.31	0.26
Bukoto 2	14.81	0.74	18.70	**1.72**	5.38	0.22	0.43	0.36
Nabukalu	14.81	0.74	14.65	**1.34**	5.38	0.22	0.19	0.16
Abdu	11.56	0.94	20.74	**1.90**	2.70	0.44	0.69	0.57
SP4306K	11.56	0.94	12.91	**1.18**	2.70	0.44	0.60	0.50
Kikoni	11.56	0.94	5.56	0.51	2.70	0.44	0.02	0.01
SP1414R	11.56	0.94	15.18	**1.39**	2.70	0.44	0.34	0.28
Sentamu	11.56	0.94	7.82	0.72	2.70	0.44	0.10	0.08
Kunya	10.90	1.00	23.60	**2.17**	2.70	0.44	0.76	0.63
SP1212R	11.56	0.94	8.29	0.76	2.70	0.44	0.10	0.09
Kibumbiro	14.81	0.74	34.67	**3.18**	5.38	0.22	1.13	**0.94**
Kabaale	11.56	0.94	14.70	**1.35**	2.70	0.44	0.94	0.78
Nababirye	10.90	1.00	6.80	0.62	2.70	0.44	0.19	0.16
SP1010R	11.56	0.94	10.46	**0.96**	2.70	0.44	0.15	0.13
Nsambya	11.56	0.94	10.18	**0.93**	2.70	0.44	0.32	0.27
Kapeke	11.56	0.94	34.88	**3.20**	2.70	0.44	0.80	0.67
Yusuf	11.56	0.94	9.90	0.91	2.70	0.44	0.35	0.29
Kasanvu	11.56	0.94	2.94	0.27	2.70	0.44	0.05	0.04
Nabagereka	19.34	0.56	8.40	0.77	5.38	0.22	0.38	0.32
Barracks	11.56	0.94	6.23	0.57	2.70	0.44	0.23	0.19
SP5003C	14.81	0.74	17.74	**1.63**	5.38	0.22	0.63	0.52

## Data Availability

The data used to support the findings of this study are available from the corresponding author upon request.
